# Conjunctival Lymphangiectasia - case report

**DOI:** 10.22336/rjo.2022.65

**Published:** 2022

**Authors:** Andreea-Petra Cristea, Lorina Tabita Petrescu, Cristina Stan

**Affiliations:** * Department of Ophthalmology, Emergency County Hospital, Cluj-Napoca, Cluj, Romania; ** Department of Ophthalmology “Iuliu Haţieganu” University of Medicine and Pharmacy Cluj-Napoca, Cluj, Romania

**Keywords:** lymphangiectasia, conjunctiva, cystic lesions

## Abstract

Conjunctival lymphangiectasia is a rare pathology that represents the enlargement of the lymphatic vessels localized in the conjunctiva. Patients may be asymptomatic or experience symptoms such as foreign body sensation, congestion, irritation, dryness, and blurry vision.

There are various methods of therapy for patients with severe and symptomatic conjunctival lymphangiectasia. Surgical excision has the lowest rates of recurrence.

We present a case of a 24-year-old woman with conjunctival lymphangiectasia and a history of left lower limb enlargement and bilaterally enlarged submandibular and upper jugular lymph nodes without an identifiable cause, who presented to the ophthalmology clinic accusing ocular discomfort, foreign body sensation and transparent conjunctival cystic lesions in the left eye for the last five months.

**Abbreviations:** OD = right eye, OS = left eye, OCT = optical coherence tomography, VEGF = vascular endothelial growth factor

## Introduction

The eye’s lymphatic system is present at the conjunctiva, lacrimal glands, and optic nerve level. Therefore, any disruption of its function will automatically lead to eye changes.

Lymphangiectasia is a rare condition defined as the pathological dilation of the lymphatic ducts, with secondary fluid retention in the interstitial space. The disease can be primary or secondary. The former appears due to the congenital developmental disorder and is associated with various systemic pathologies such as Fabry disease, Turner syndrome, and Nonne-Milroy-Miege disease. The latter is most likely caused by the obstruction of the lymphatic vessel due to various motives: irradiation, trauma, inflammation, and ocular surgery [**1**].

Conjunctival lymphangiectasia may have a diffuse distribution, with conjunctival chemosis, or a localized one with a “string of pearls” appearance. Localization may be unilateral or bilateral, which is more frequent in association with a systemic disease.

Some patients complain of symptoms such as: foreign body sensation, congestion, irritation, dryness, and decreased visual acuity.

Most of the time, conjunctival lymphangiectasia resolves spontaneously without treatment. Severe or symptomatic cases may need treatment. Various therapy types are available, such as liquid nitrogen cryotherapy, carbon dioxide (CO2) laser, or surgical excision. Although surgical excision is more invasive, it has a much lower recurrence rate [**2**].

## Case report

**Fig. 1:**
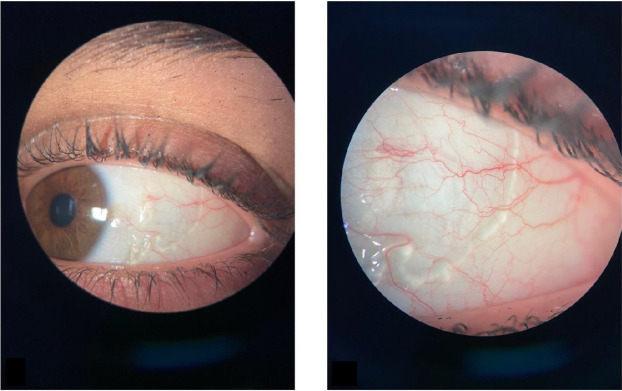
Slit lamp examination of the left eye: sausage-shaped clear channels pattern

A 24-year-old woman presented to the ophthalmology clinic accusing a 5-month history of ocular discomfort, foreign body sensation, and transparent conjunctival cystic lesions in the left eye. The initial diagnosis was conjunctivitis with the initiation of the topical treatment (steroids and antibiotics), but without a favorable response. However, several months later, due to the localized aspect of the conjunctival changes, a subconjunctival worm was suspected, and the patient was referred to our clinic. The visual acuity measured with the Snellen chart was 1 in both eyes without the need for correction. The slit lamp examination revealed sausage-like conjunctival cystic lesions in the left eye (OS) (**Fig. 1**) and no changes in the right eye (OD). We observed that the conjunctival lesions were immobile and transparent (the sclera could be seen clearly), and there was no chemosis or conjunctival congestion. 

The posterior pole examination was within normal limits. The patient’s intraocular pressure was 14 mmHg in the right eye and 13 mmHg in the left one. The patient’s history revealed allergic conjunctivitis triggered by exposure to pollen and previous allergic reactions to chlorine products and penicillin. Two years prior, the patient experienced an abnormal painless circumferential enlargement of approximately 2 cm diameter of the entire left lower limb without an identifiable underlying pathology and spontaneous remission. The possibility of a venous thrombosis was ruled out following a cardiovascular evaluation. At the beginning of the current year, a cervical CT scan was performed after the discovery of bilaterally enlarged submandibular and upper jugular lymph nodes, the examination confirming their benign nature. The patient is a smoker and denies any exposure to other toxics. 

**Fig. 2:**
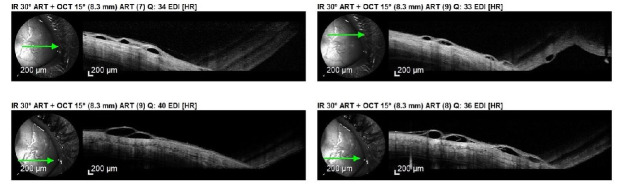
Anterior segment OCT: Cystic fluid filled lesions of different calibre in the sub-conjunctival space

We decided to perform an anterior segment optical coherence tomography (OCT), which revealed cystic fluid-filled lesions of different caliber in the sub-conjunctival space, suggestive of dilated lymphatic vessels (**Fig. 2**).

Our final diagnosis was: OS Conjunctival Lymphangiectasia.

We suggested the surgical excision of the lesion, but the patient refused.

## Discussions

As mentioned above, conjunctival lymphangiectasia is a rare disorder representing the distension of the lymphatic vessels either primarily or secondary to inflammation or infiltration. The alteration of the lymphoid system can occur secondary to local trauma such as surgery or radiotherapy in the head and neck area [**3**]. In our case, the patient did not have any history of ocular surgical interventions, nor has she ever received radiation therapy. The only abnormal aspect worth mentioning was the bilateral painless enlargement of the submandibular and upper jugular lymph nodes, for which a cause could not be identified. 

There are various ways in which lymphangiectasia can clinically present, such as thickened conjunctiva with cysts, chemosis, the “string of pearls” conjunctival swelling, or sausage-shaped clear channels. In cases that present as localized chemosis, the first diagnosis of exclusion that should be considered is allergic conjunctivitis [**3**]. Our patient is known for having seasonal allergic conjunctivitis and describes those episodes as presenting with significant itching and eye discharge, notably different compared to the moment of presentation to our clinic, when the foreign body sensation and transparent conjunctival cystic lesions were the main ocular complaints. At the clinical exam, conjunctival hyperemia, chemosis and follicles were absent. Abnormal accumulation of fluid in the interstitial space can occur in pathologies that are characterized by hypoproteinemia [**3**]. We mentioned that our patient was not aware of any chronic condition.

Recent literature data associates conjunctival lymphangiectasia with Fabry disease, the link between the two rare entities being the altered lymphatic system. Even though the most common ocular clinical finding in people suffering from this lysosomal storage disorder is cornea verticillata, the presence of conjunctival alteration should not be disregarded [**1**]. Although in the general population the prevalence of conjunctival lymphangiectasia is only 1%, the percentage was proven to be significantly higher in patients with hereditary transthyretin amyloidosis. The ocular surface change is currently regarded as a possible severity biomarker [**4**]. 

The most relevant differential diagnosis that we must consider when facing a patient with this clinical presentation is conjunctival lymphangioma, a benign tumor with progressive growth. The presence of lymphangiomas in other locations can be relevant for the diagnosis, but the most conclusive examination is the histopathological evaluation. These cases respond well to surgical excision, even though some of them may resolve spontaneously [**5**]. At the clinical examination, our patient did not present lymphangiomas in any other part of the body and the lesion did not increase in size since its appearance, 5 months before the eye examination. 

Multilocular inclusion cysts may also be considered, since they present as clear fluid-filled round lesions of the conjunctiva. They can arise spontaneously or secondary to local irritation. A clear diagnosis can only be made after a biopsy, and surgical resection is the treatment of choice when needed [**3**]. Another differential diagnosis worth mentioning is the subconjunctival worm, which was initially suspected in our case. This may be found in people who traveled in tropical countries, but, contrary to our patient, usually manifests with conjunctival chemosis and congestion. Patients also complain of a “crawling” sensation in the eye. The histopathological examination establishes the diagnosis [**6**].

The main method of diagnosis in conjunctival lymphangiectasia is histopathological examination. We could not perform it because our patient refused any interventional procedure. However, we performed an anterior segment OCT, which was tremendously helpful for the non-invasive diagnosis of this pathology. The obtained image was characterized by the presence of hyporeflective lesions with different sizes, representing the fluid-filled dilated lymphatic vessels [**7**]. Our patient presented the textbook description of the lesions, therefore, leading us to the correct diagnosis.

Treatment options for patients with conjunctival lymphangiectasia include surgical excision, radio wave electro‐ablation, cryotherapy, and anti-vascular endothelial growth factor (anti-VEGF) subconjunctival injections. The resection of the lesion and the suturing of the remaining tissue with an 8.0 Vicryl is the option presented by Seven et al. in a case report published in 2018 [**8**]. Excision followed by conjunctival autograft or amniotic membrane transplant was also described by Welch and col., with good post-intervention results and reduced recurrence rates [**3**]. The option of liquid nitrogen cryotherapy was described in an article published in 2009, in which recurrences occurred, but were favorably treated with a second cryotherapy session with beneficial outcomes [**9**]. A single dose of subconjunctival injection with Avastin was proven to be effective in treating a patient with conjunctival lymphangiectasia, with VEGF playing significant roles in lymphangiogenesis [**10**]. The most recent treatment option is represented by conjunctival ablation with high-frequency radiowaves, thought to be a faster and safer option compared to the classic excision [**11**]. 

In our case, the patient refused the mentioned treatment options for the current moment and is kept under observation.

## Conclusion

Conjunctival lymphangiectasia is often misdiagnosed because it is confused with other causes of conjunctival swelling. Precise anamnesis and examinations are necessary for an accurate diagnosis and effective treatment.
